# Seizures Related to Neurocysticercosis and Cocaine Use

**DOI:** 10.7759/cureus.22488

**Published:** 2022-02-22

**Authors:** Benjamin S Daines, Katherine G Holder, Balaji Mohanakrishnan, James W Walker

**Affiliations:** 1 Internal Medicine, Texas Tech University Health Sciences Center, Amarillo, USA

**Keywords:** drug-induced seizure, taenia solium, infectious and parasitic diseases, seizure, cocaine use, neurocysticercosis

## Abstract

Neurocysticercosis (NCC) is an infection of the central nervous system with *Taenia solium* cysts that most commonly results in seizures. In stable patients without recent symptoms, these seizures may be provoked by seizure threshold-lowering drugs such as cocaine. This case details a 38-year-old male with a past medical history of epilepsy presenting with seizures due to comorbid NCC and cocaine use. This case was complicated by the lack of available information regarding the patient’s past medical history and medication use. We highlight the importance of obtaining a full work-up, including brain imaging, to provide optimal treatment for patients with seizures despite a history of drug use.

## Introduction

Neurocysticercosis (NCC) is the most common helminthic central nervous system infection in humans and is a common cause of epilepsy throughout South America [1,2]. While NCC is a rare diagnosis in the United States with an estimated incidence of 0.2 to 0.6 cases per 100,000, it disproportionately impacts Hispanic populations in the United States with an incidence up to 5.8 cases per 100,000 [3]. With a growing Hispanic community in the United States, NCC has become an increasingly important clinical consideration due to significant associated morbidity via seizures, hydrocephalus, and cerebral edema [4]. Early recognition and treatment of NCC with cysticidal drug therapy can reduce recurrence of seizures and aid in resolution of parenchymal cysts [5].

Cocaine use can also result in seizures or exacerbate an underlying seizure disorder by lowering the seizure threshold due to indirect sympathomimetic effects [6]. Patients in the emergency department who had used cocaine presented due to seizures in nearly 10% of cases [7]. While cases of cocaine-induced seizures have decreased, cocaine use is still an important consideration in a patient presenting with seizures [8]. Currently, there are no peer-reviewed cases in the literature describing comorbid NCC and cocaine use. Here, we present a case of a 38-year-old male with seizures due to NCC and cocaine use.

## Case presentation

A 38-year-old male with a past medical history of epilepsy presented after two episodes of seizures 3 hours apart on the day of presentation. The seizures were witnessed by family. Each episode lasted approximately 5 minutes and resulted in loss of consciousness with post-ictal confusion. The patient denied nausea, vomiting, bowel changes, chest pain, shortness of breath, paresthesia, weakness, vision changes, and headache. He endorsed some fatigue. The patient was asleep prior to the episodes and had no recent changes in physical activity, eating, drinking, or stressful inciting events. He denied recent sick contacts and travel. He endorsed drinking alcohol 1-2 times weekly but denied tobacco and recreational drug use. One year ago, the patient moved to the United States from Mexico where he had lived his entire life. He stopped taking his seizure medication for epilepsy one year ago. He does not know what seizure medication he was taking nor any details of his epilepsy diagnosis from childhood. He says he has not had a seizure since he was a child. He endorsed no family history of epilepsy or other medical conditions.

On physical examination, there were no focal neurological deficits. Cranial nerves II through XII were intact with normal sensation and strength in all extremities bilaterally. Deep tendon reflexes were 2+ bilaterally. The patient was alert and oriented to person, place, and time. Vital signs were stable and initial laboratory values in Table 1 revealed mild leukocytosis without eosinophilia. Urine drug screen was positive for cocaine. When this result was brought up, the patient admitted to using cocaine a few times in the past, but not habitually. He said his most recent cocaine use was a few days ago. The previous cocaine use with normal physical examination findings indicated a likely drug-induced seizure, but additional imaging was ordered due to the unknown etiology of the patient’s childhood epilepsy diagnosis.

**Table 1:** Laboratory Results WBC: white blood cells; ALP: alkaline phosphatase; ALT: alanine transaminase; AST: aspartate transaminase

Investigation	Value	Reference Range
WBC (k/µL)	12.8	4.8-10.8
Hemoglobin (g/dL)	14.8	14.0-18.0
Eosinophils (%)	0.5	0.0-5.0
Platelets (k/µL)	251	150-400
Sodium (mmol/L)	140	135-145
Potassium (mmol/L)	4.0	3.5-5.0
Chloride (mmol/L)	109	98-107
Bicarbonate (mmol/L)	20	21-31
ALP (U/L)	67	25-100
ALT (U/L)	40	10-40
AST (U/L)	31	12-38

**Figure 1:**
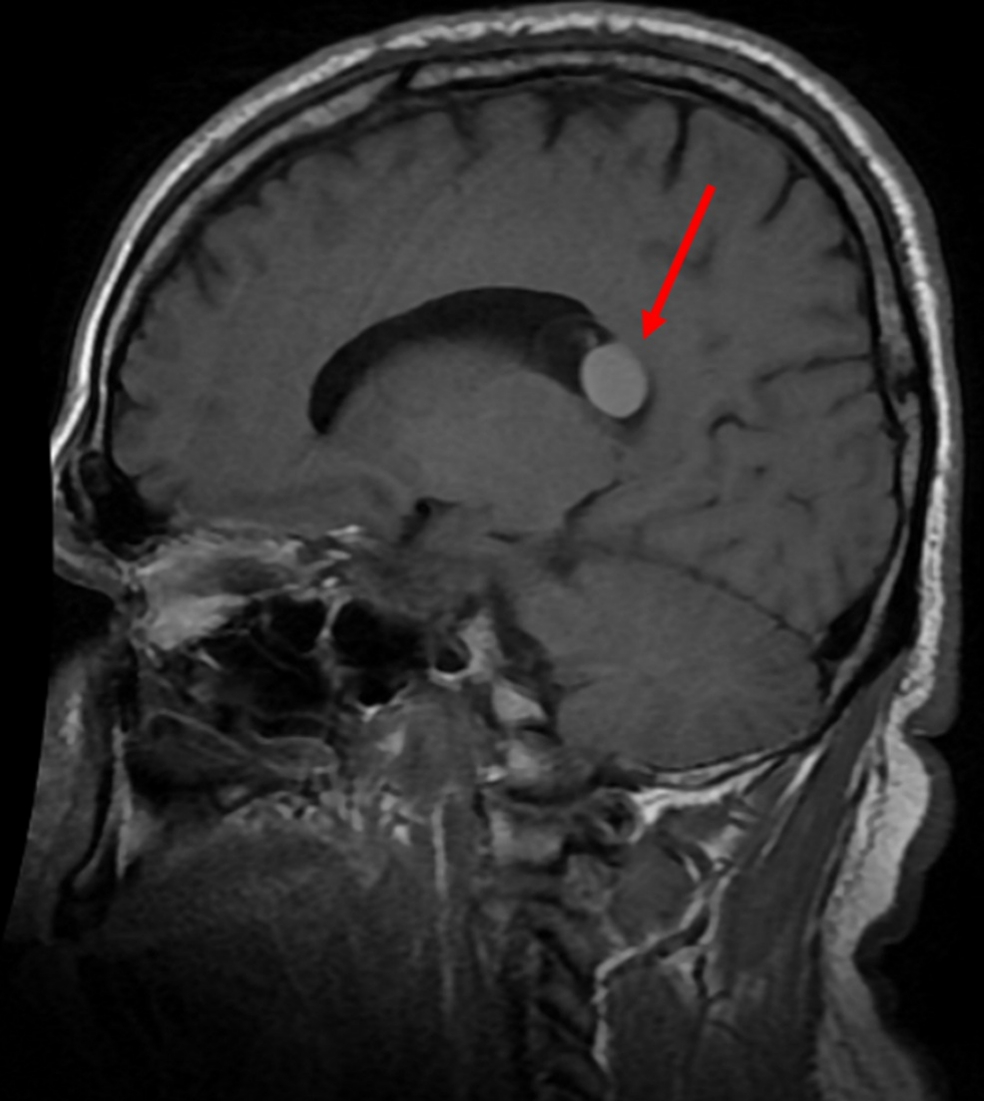
Sagittal T1-weighted brain MRI demonstrating non-enhancing hyperintense lesion.

**Figure 2:**
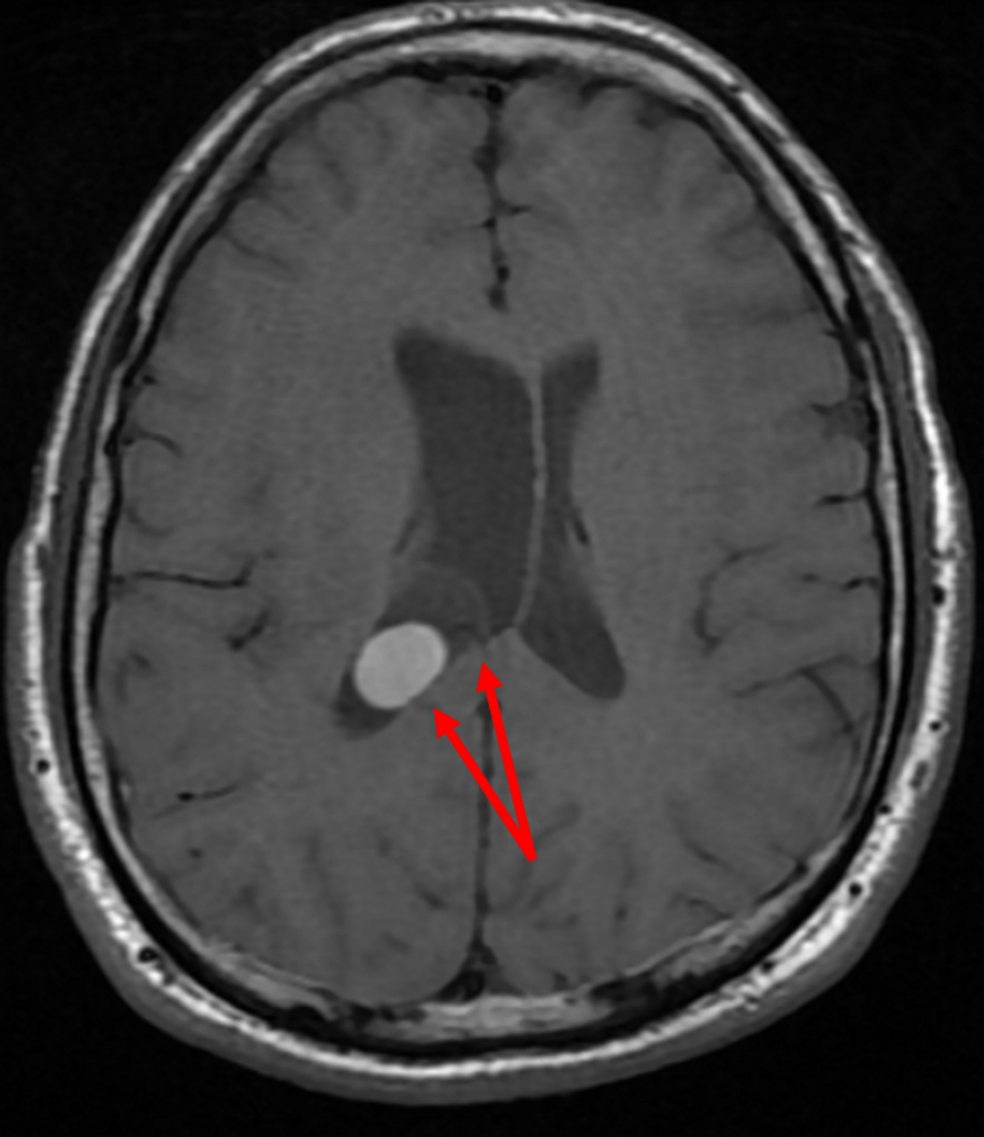
Axial T1-weighted brain MRI demonstrating hyperintense lesion within larger cystic lesion and enlargement of the right ventricle.

Brain MRI demonstrated a non-enhancing T1 hyperintense 1.7 cm diameter lesion (Figure 1) within a larger cystic 2.6 cm diameter lesion in the atrium of the right lateral ventricle adherent to the choroid (Figure 2). There was asymmetric enlargement of the right ventricle. These MRI findings combined with the previous diagnosis of epilepsy and social history of growing up in Mexico indicated a clinical diagnosis of NCC. The patient was subsequently started on lorazepam 2 mg IV as needed and levetiracetam 500 mg IV every 12 hours while inpatient for seizure prevention. Neurosurgery determined surgical intervention was unnecessary at the time and requested a follow-up MRI in six months to determine cyst stability. Infectious disease recommended a 14-day and 21-day course of albendazole and dexamethasone, respectively, with neurology follow-up for seizure control. The patient appeared clinically well with no new seizures or neurological changes at 24 hours, so they were discharged on albendazole 600 mg twice a day for 14 days, dexamethasone 2 mg three times a day for 21 days, and levetiracetam 500 mg twice a day until neurology follow-up. The patient was advised to stop recreational drug use to help prevent seizure recurrence. Follow-up imaging and neurology appointments were scheduled.

## Discussion

NCC is a common cause of seizures worldwide and occurs due to ingestion of eggs from the cestode *Taenia solium* [1]. These eggs are transmitted fecal-orally from a human carrier of the tapeworm [1]. *T. solium* is responsible for both cysticercosis via ingestion of eggs and taeniasis, a mild gastrointestinal infection via ingestion of cysts from undercooked pork [1]. NCC is cysticercosis that develops in the central nervous system. The variety in number, size, stage, and location of cysts along with differences in host immune response results in varied pathophysiologic effects [9]. While vestibular cysticerci tend to result in little inflammation, colloidal and meningeal cysticerci can cause intense inflammatory responses resulting in gliosis and edema [9]. The chronic nature of NCC due to the persistence of cysts for years along with a varied disease progression makes diagnosis a significant challenge [9].

Symptomatic NCC most commonly presents with seizures, but many will present with hydrocephalus, focal neurologic deficits, or headache [10]. Diagnosis may be a challenge due to non-specific presentation coupled with difficulty in obtaining a biopsy specimen for histopathologic analysis [9]. Initial diagnosis begins with neuroimaging via CT and MRI [9]. Visualization of several cysts displaying “hole-with-dot” patterns indicative of the tapeworm scolex can confirm a diagnosis of NCC [9,11]. Locating such an image for diagnosis is often unattainable due to variability in stage of cyst involution [9]. Cysticerci often appear as non-enhancing hyperdense lesions [9]. MRI with fluid-attenuated inversion recovery tends to better visualize intraventricular cysts, such as in this case [11]. Gold standard for diagnosis with 98% sensitivity and 100% specificity is an enzyme-linked immunoelectrotransfer blot (EITB) for *T. solium* antibodies in serum [12].

While EITB was not obtained in this case, a clinical diagnosis could be made with characteristic imaging findings alongside the patient’s social history. Epidemiologic factors play a critical role in the diagnosis of NCC. NCC is endemic to South America, parts of Central America, and Asia [3]. NCC rates in autopsies from these countries are as high as 3.6% [9]. In Mexico, records from a large neurologic hospital found an NCC rate of 2.5% [13]. With changes in immigration patterns, 90% of NCC diagnoses in the United States and Europe are Central American immigrants [3]. In this case, the patient was an immigrant from Mexico with epilepsy of unknown origin, so NCC was an important consideration. Diagnosis should be supplemented by history of immigration from endemic areas or contacts with known carriers of *T. solium*.

No previous literature has linked NCC-induced seizures to cocaine use. While the patient in this case endorsed a lack of seizures since childhood with and without their anti-seizure medication, the recent cocaine use likely contributed to the new-onset seizures. Cocaine can lower the seizure threshold via indirect sympathomimetic effects [6]. While most cocaine-induced seizures appear to occur within hours of use, cocaine metabolites can persist for days before clearance [14,15]. This persistent adrenergic hyperactivity may lower the seizure threshold for an extended period. Previous studies investigating the effects of cocaine use on grey and white matter have shown inconsistent results and cocaine appears to have no significant impact on overall brain structure [16]. Further investigation is necessary to determine the impact of cocaine and brain masses like NCC on provocation of seizures.

Typical treatment of NCC typically consists of cysticidal drug therapy, anti-seizure medication, and anti-inflammatories. Albendazole (15 mg/kg/day) has been identified as the superior cysticidal therapy for reducing the burden of cystic brain lesions due to NCC [17]. Praziquantel (50 mg/kg/day) is another available therapy, albeit a less efficacious option for reducing cysts [17]. For seizure prevention, a first-line anti-epileptic, such as levetiracetam in this case, is adequate [3]. The length of anti-epileptic treatment is a continuing matter of debate with a previous study finding 50% of patients with successfully treated brain cysticerci experiencing seizures after withdrawal of anti-epileptics [18]. The patient in this case was discharged on levetiracetam to be adjusted at the discretion of his neurologist. Anti-inflammatories, such as steroids, have shown efficacy in reducing seizures due to cyst degeneration via albendazole or praziquantel [19]. In this case, dexamethasone (0.1 mg/kg/day) was used for the duration of cysticidal therapy to successfully prevent seizures.

## Conclusions

This patient presented with a seizure and evidence of comorbid NCC and cocaine use. While NCC is exceedingly rare in the United States, it should be an important consideration in any patient from an NCC-endemic region presenting with seizures of unknown origin. Despite initial urine drug screen findings, thorough work-up including brain imaging is necessary to appropriately diagnose and treat patients with seizures. Treatment with cysticidal therapy and drug cessation counseling can prevent subsequent seizures and significantly improve patient symptoms.
